# Supplementary figures and images for: Radiomics machine learning study with a small sample size: Single random training-test set split may lead to unreliable results
Source: PLoS One. 2021 Aug 12;16(8):e0256152. doi: 10.1371/journal.pone.0256152 (PMC8360533; doi:10.1371/journal.pone.0256152)

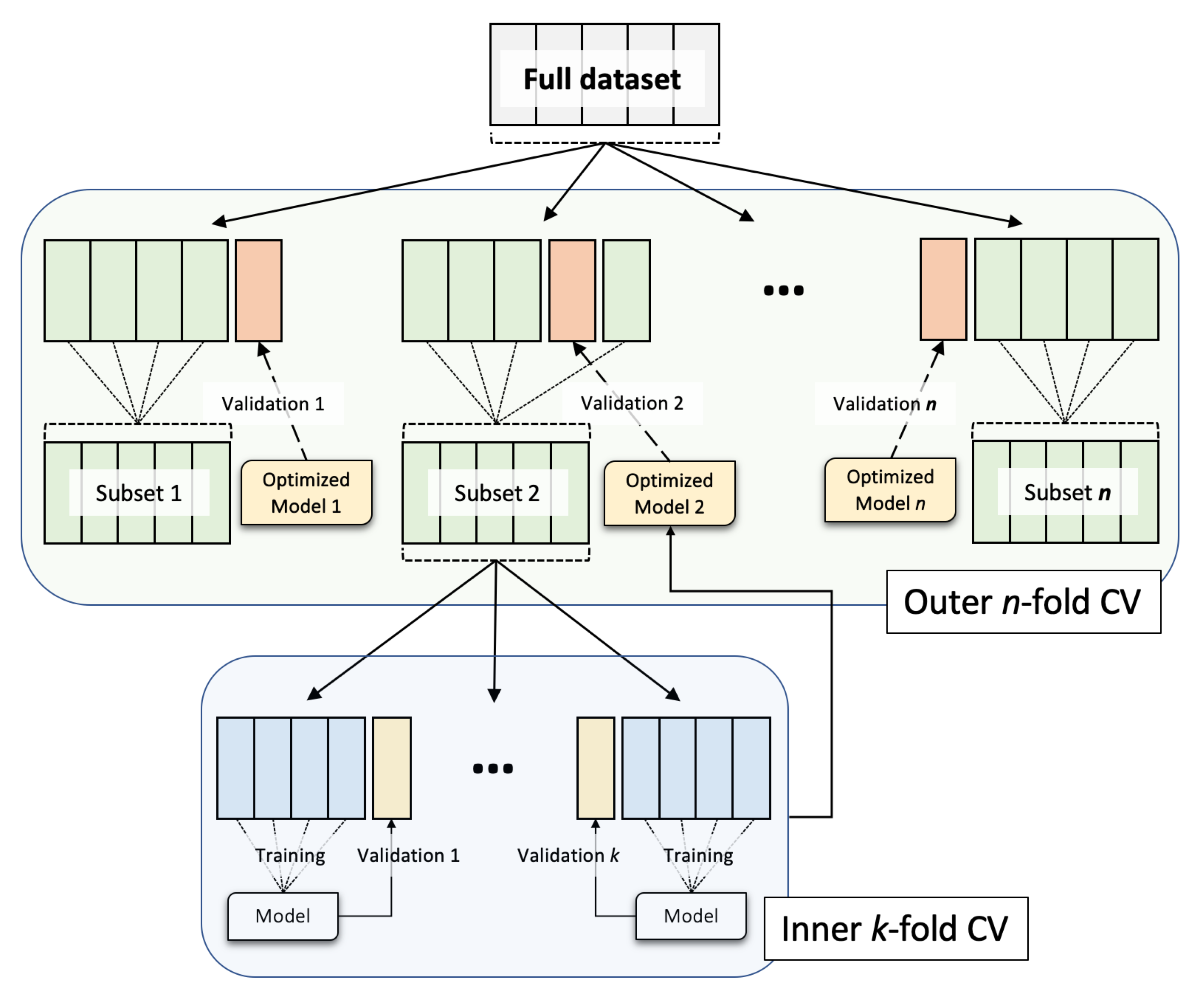

Supplement: S1 Fig — The inner loop is responsible for model selection and hyperparameter tuning, while the outer loop is used for error estimation. (TIF) [file pone.0256152.s001.tif]
