# Supplementary material for: Radiomics machine learning study with a small sample size: Single random training-test set split may lead to unreliable results
Source: PLoS One. 2021 Aug 12;16(8):e0256152. doi: 10.1371/journal.pone.0256152 (PMC8360533; doi:10.1371/journal.pone.0256152)
Supplement: Attachment — Submitted filename: Response to Reviewers.docx [file pone.0256152.s002.docx]

**Journal Requirements:**

When submitting your revision, we need you to address these additional requirements.

 1. Please ensure that your manuscript meets PLOS ONE's style requirements, including those for file naming.

>>> Using the sample files, we revised the manuscript so that its style matches the PLOS ONE’s style requirement.

2. Thank you for including your ethics statement:

"This retrospective study (2020-2996-001 ) was approved by the Institutional Review Board, and the informed consent from the patients was waived."
Please amend your current ethics statement to include the full name of the ethics committee/institutional review board(s) that approved your specific study.
Once you have amended this/these statement(s) in the Methods section of the manuscript, please add the same text to the “Ethics Statement” field of the submission form (via “Edit Submission”).

>>> We revised the sentence so that it includes the full name of our IRB.

3. In your Data Availability statement, you have not specified where the minimal data set underlying the results described in your manuscript can be found. PLOS defines a study's minimal data set as the underlying data used to reach the conclusions drawn in the manuscript and any additional data required to replicate the reported study findings in their entirety. All PLOS journals require that the minimal data set be made fully available. For more information about our data policy, please see <http://journals.plos.org/plosone/s/data-availability>.

Upon re-submitting your revised manuscript, please upload your study’s minimal underlying data set as either Supporting Information files or to a stable, public repository and include the relevant URLs, DOIs, or accession numbers within your revised cover letter. For a list of acceptable repositories, please see http://journals.plos.org/plosone/s/data-availability#loc-recommended-repositories. Any potentially identifying patient information must be fully anonymized.

We will update your Data Availability statement to reflect the information you provide in your cover letter.

 >>> We have uploaded all the data and codes used in the study on our GitHub repository, after de-identification.

4. Please include captions for your Supporting Information files at the end of your manuscript, and update any in-text citations to match accordingly. Please see our Supporting Information guidelines for more information: [http://journals.plos.org/plosone/s/supporting-information](about:blank).

>>> We have added the captions for the Supporting Information at the end of our manuscript.

5. Please upload a copy of Figure S1, to which you refer in your text on page 7. If the figure is no longer to be included as part of the submission please remove all reference to it within the text.

 >>> We have uploaded Figure S1.

6. Please include a copy of Table S2 which you refer to in your text on page 10.

>>> In the revised manuscript, Tables S1 and S2 are no longer included.

**Reviewers’ comments:**

Reviewer #1:

1. The keywords are missing in the abstract.

>>> We added five keywords (words included in the title were not selected as keywords): prediction model, validation, data splitting, glioblastoma, brain metastasis

2. The main contribution of the work must be properly framed in the introduction.

>>> Thanks to the reviewers’ valuable comments and suggestions, we extensively revised the manuscript (We have practically rewritten the entire manuscript), inspired by the reviewers’ comments and suggestions. In the revised Introduction, the main contribution of our work is summarized as follows:

“Despite its importance, the stability issue has received little attention in the radiomics ML community, as the main focus has been on model performance. In the field of radiomics-based ML, to the best of our knowledge, no study has investigated the size of the variabilities caused by random data splitting using real-world radiomics data. Specifically, we were interested in exploring how widely the radiomics ML results—model performance estimated in training and that obtained in testing—can vary depending on how a dataset is split into training and test sets, which could be used as caveats when conducting radiomics ML studies and interpreting the results.

3. The introduction must highlight the actual problem the authors tried to solve.

>>> In the revised Introduction, the actual problem we tried to solve is highlighted; the following is excerpted from the revised manuscript:

“… many ML studies with medical image data, including radiomics-based ML, are often conducted in a small group of patients… but still reporting promising predictive accuracies… external validation was missing in 81–96% of published radiomics-based studies… In the absence of external validation data, one of the most common strategies to prove the validity of a model’s performance is randomly splitting an available dataset into training and test sets… random data splitting, by reducing sample sizes and adding more randomness, increases the errors in estimated model performance and the instability of validation results, especially with small sample sizes, which may lead to unstable and suboptimal model development, and overly optimistic performance estimation… the purpose of this study was to investigate how splitting a dataset randomly into training and test sets affects the ML model’s estimated performance and its difference from internally validated performance using real-world brain tumor radiomics.”

4. The literature review is very very weak in the article; author needs to append more quality works from 2019-2021.

>>> Thank you for your comment. We have thoroughly reviewed articles on machine learning and model evaluation methods and updated several references accordingly. Only few relevant methodology papers have been published in recent years, which were still very informative and helped us to improve our manuscript. The following are the papers published within 5 years that we have newly cited in the revised manuscript:

5. Ramspek CL, Jager KJ, Dekker FW, Zoccali C, Diepen M van. External validation of prognostic models: what, why, how, when and where? Clin Kidney J. 2021;14: 49–58.

6. Won SY, Park YW, Ahn SS, Moon JH, Kim EH, Kang S-G, et al. Quality assessment of meningioma radiomics studies: Bridging the gap between exploratory research and clinical applications. Eur J Radiol. 2021;138: 109673.

7. Won SY, Park YW, Park M, Ahn SS, Kim J, Lee S-K. Quality Reporting of Radiomics Analysis in Mild Cognitive Impairment and Alzheimer’s Disease: A Roadmap for Moving Forward. Korean J Radiol. 2020;21: 1345.

20. Jiang Y, Krishnan D, Mobahi H, Bengio S. Predicting the Generalization Gap in Deep Networks with Margin Distributions. arXiv:1810.00113v2 [Preprint]. 2018 [cited 2021 May 20]. Available from: https://arxiv.org/abs/1810.00113v2

22. Austin PC, Steyerberg EW. Events per variable (EPV) and the relative performance of different strategies for estimating the out-of-sample validity of logistic regression models. Stat Methods Med Res. 2017;26: 796–808.

24. Varoquaux G. Cross-validation failure: Small sample sizes lead to large error bars. Neuroimage. 2018;180: 68–77.

27. Steyerberg EW, Harrell FE. Prediction models need appropriate internal, internal–external, and external validation. J Clin Epidemiol. 2016;69: 245–247.

28. Siontis GCM, Ioannidis JPA. Response to letter by Forike et al.: more rigorous, not less, external validation is needed. J Clin Epidemiol. 2016;69: 250–251.

29. Martens FK, Kers JG, Janssens ACJW. External validation is only needed when prediction models are worth it (Letter commenting on: J Clin Epidemiol. 2015;68:25-34). J Clin Epidemiol. 2016;69: 249–250.

32. Ho SY, Phua K, Wong L, Goh WWB. Extensions of the External Validation for Checking Learned Model Interpretability and Generalizability. Patterns. 2020;1: 100129.

33. Olsen MK, Stechuchak KM, Steinhauser KE. Comparing internal and external validation in the discovery of qualitative treatment-subgroup effects using two small clinical trials. Contemp Clin Trials Commun. 2019;15: 100372.

5. Which datasets the authors have used is not getting clear in the Materials & Methods section, authors need to describe more about the datasets used.

>>> We have added more description of the datasets used. In addition, we have uploaded all the radiomics datasets and R/Python codes used for this study on our GitHub repository so that anyone can reproduce our results.

URL: https://github.com/Chansikan/do_not_split_small_sample

6. The paper is mathematically weak, authors need to check this issue carefully, they not show the mathematical part also.

>>> Thank you for your valuable comment. We also discussed with a biostatistician. However, we decided that delving into mathematical aspects would be beyond our capabilities (we were afraid that we would end up conveying inaccurate information) and that it would be better to focus on demonstrating the actual consequences with real-world radiomic-based ML studies. Instead, we explicitly explained this limitation in Discussion.

7. The proposed work needs to be compared with some existing works from 2019-2021 for understanding the novelty and efficiency of the proposed work.

>>> In the revised Introduction and Discussion sections, we explained what distinguishes our study from others, although only few papers have been published in recent years, as mentioned above. For examples, the following excerpted from the revised manuscript:

“In the field of radiomics-based ML, to the best of our knowledge, no study has investigated the size of the variabilities caused by random data splitting using real-world radiomics data.”

“… it is well known that the sufficient training data size depends on the complexity or difficulty of a task... However, what we attempted to demonstrate is that, ironically, the harder a radiomics ML task is, the more overly optimistic results we can obtain by random data splitting.”

“Several methods have been proposed to obtain the reliable estimates of true model performance, supported by our results that using some of these methods—nested CV or bootstrapping—could reduce the optimism in estimating model performance. However, we also found that a significant data mismatch between training and test datasets can be challenging to overcome by any technique…”

8. The discussion needs more elaboration and explanation.

>>> As mentioned above, we rewrote nearly the whole manuscript, which we believe improved the manuscript much. The revised Discussion now includes detailed explanation and interpretation of our results.

9. Only high definition images must be used in the manuscript. Figure 1,2 needs to be changed with HD images.

>>> We have changed the Figures 1 and 2 to high-resolution images and uploaded them.

Reviewer #2: The manuscript addresses the reliability of radiomics analysis when there are less samples and they are randomly splitted into training and test dataset. The study is of interest and timely.

The manuscript may be accepted for publication.

>>> We would like to express our gratitude to your positive review.

Reviewer #3: In this paper, the authors tackled an important problem of estimating the classification abilities of machine learning classification models in the case of limited ground truth data. The topic investigated in this paper is definitely worth considering, but the manuscript suffers from the following shortcomings that need to be addressed before it could be considered for publication:

1. The experiments reported in the paper should be fully reproducible. Hence, the authors should make their MRI data publicly available. If it is not possible due to any reason, the authors should explot a publicly available dataset in their experiments (such as the BraTS dataset). Similarly, I encourage the authors to make their implementation publicly available as well.

>>> We uploaded all the radiomics datasets and R/Python codes used for this study on our GitHub repository (also mentioned in the revised manuscript), so that anyone can fully reproduce our results.

URL: https://github.com/Chansikan/do_not_split_small_sample

2. The authors mentioned the accuracies reported in the literature for the investigated classification problems in the Materials and Methods (Subjects) section — for what data were these accuracies reported?

>>> Thank you for this valuable question. In the revised manuscript, we explained in more detail the characteristics of the datasets from which the accuracies were obtained, by describing the MRI sequences used, and citing a reference separately for each reported accuracy so that readers can refer to it if they’re interested in more information.

3. All entities in the paper should be self-contained — to this end, the authors should expand the captions of their figures and include all the details that will make the figures possible to understand without diving into text. Similarly, please discuss the exploited methods in detail (e.g., De Long method).

>>> Thank you for pointing this out. We added detailed explanation in the figure legends so that the figures can be understood without diving into the text.

>>> Also, we revised the manuscript so that readers can understand the methods (i.e., CV with repetitions, nested CV, bootstrap, De Long method) used in our study by reading the text or referring to the references. Furthermore, since now we have made our codes publicly available, anyone can actually implement those methods by themselves.

4. Although the manuscript reads well in general, I spotted a typo (imagining).

>>> We have corrected the typo and some other awkward phrases.
