# Supplementary material for: Radiomics machine learning study with a small sample size: Single random training-test set split may lead to unreliable results
Source: PLoS One. 2021 Aug 12;16(8):e0256152. doi: 10.1371/journal.pone.0256152 (PMC8360533; doi:10.1371/journal.pone.0256152)
Supplement: Attachment — Submitted filename: Respone to Reviewers.docx [file pone.0256152.s003.docx]

Reviewer #1: Although the manuscript is revised by the authors as per previous comments, but still certain points needs to be considered for modification before considering the paper which is as follows:
1. The dataset citation is still missing in the paper.

>>> Thank you for your comment. All the raw datasets and (R and Python) codes can be found in a publicly open repository which we have cited in our paper as follows:

“All the datasets and codes used in this study can be found on our GitHub repository [13].” (Page 5 Line 104)

13. An C. GutHub page: do not split small samples; 2021 [cited 30 May 2021]. Available from: <https://github.com/Chansikan/do_not_split_small_sample>)

2. The authors are suggested to make a tabular format for comparison of the proposed with existing works.

>>> Thank you for your valuable comment. We have thoroughly searched in PubMed MEDLINE and EMBASE databases to identify papers with similar purposes to ours. We believe we tried our best. However, unfortunately, we could not find a study that had the similar objective as ours (to prove that random splitting of training and test sets may lead to unreliable results) in the radiomics machine learning filed. In fields other than radiomics, we could not find a paper with the same purpose either, although there were papers where it was only secondary aim or side results, which we already cited in the manuscript [10, 23, 24, 25, 28]. If the reviewer suggests which papers can be compared to the current work, we would be happy to review them and make a tabular format for comparison. Again, we thank you for your comment to improve the overall quality of our manuscript.

3. The conclusion is very short, authors need to revise it highlighting the research result and the possible future work.

>>> As you have advised, we have extended the conclusion and highlighted the research result, and mentioned possible future work in the revised manuscript as follows:

“When a sample size is not sufficient for a radiomics ML task, the model’s performance estimated in training and that obtained in testing may vary widely between different training-test set pairs. Therefore, a single random split of a dataset into training and test sets may lead to an unreliable report of the estimated model performance and generalization gap. Furthermore, since the variability of generalization gap tends to be wider with smaller sample sizes and more difficult tasks, ironically, the harder a radiomics ML task is, the more overly optimistic results we can obtain by random data splitting. Therefore, we advise against splitting a small dataset into training and test sets and recommend reducing the optimism in estimating model performance by using bootstrapping, nested CV or other techniques to better predict generalization gap, when external validation is not performed. Future study with real-world data other than brain MRI radiomics is warranted to further investigate the impact of random data splitting on ML study results.”

Reviewer #3: I am happy to see that the authors have addressed my concerns. I would, however, still encourage the authors to present the De Long method in more detail in the main body of the manuscript.

>>> Thank you for your comment. As suggested, first we tried to summarize how the method works in several sentences, but it would take at least a couple of paragraphs, which we don’t think is appropriate. Then we tried to explain the method in a separate supplementary material. However, it would end up just copying (with some modification) the contents from a review paper. Therefore, we decided to cite an excellent, easy-to-follow review paper that explains in detail the De Long method and demonstrates how it is calculated. In the main body the manuscript, we added the following sentence with citing the paper: “Interested readers can also find a detailed explanation on how to implement the DeLong method in a review paper [12].”

[12] Hanley JA, Hajian-Tilaki KO. Sampling variability of nonparametric estimates of the areas under receiver operating characteristic curves: An update. Acad Radiol. 1997;4(1):49-58. doi:10.1016/s1076-6332(97)80161-4
